# Supplementary material for: Probiotics for Reduction of Examination Stress in Students (PRESS) study: A randomized, double-blind, placebo-controlled trial of the probiotic Lacticaseibacillus rhamnosus HN001
Source: PLoS One. 2022 Jun 8;17(6):e0267778. doi: 10.1371/journal.pone.0267778 (PMC9176810; doi:10.1371/journal.pone.0267778)
Supplement: S1 File — Deidentified dataset. (DOCX) [file pone.0267778.s001.docx]

**Probiotics for Reduction of Exam Stress in Students (PRESS) Study: A randomised, double-blind, placebo-controlled trial**

**BACKGROUND**

There is increasing awareness of stress and the detrimental effect it has on both psychological wellbeing and physical health. This awareness has prompted interest in interventions that are designed to help people prevent stress, manage stress when it does occur and promote wellbeing. Many of these interventions in educational and employment settings include a focus on lifestyle improvements in exercise and diet.

The microbiota in the human gut are a colony of microbes (bacteria, viruses and fungi) that play an important role in physiological and biochemical processes in the body. Increasingly the balance between beneficial and pathological microbiota in the gut has been the subject of interest for a range of health conditions including psychological wellbeing. The microbiota gut brain axis refers to multiple bidirectional pathways that exist between the microbes in the gut and the brain. These pathways include the stress response system and immune system, both pathways which have been linked to stress, anxiety and depression [1-3].

There is evidence that disruption to the gut microbial balance influences the way the stress-response system functions in the body [4]. Stress in turn can alter the balance of microbiota in the gut This is the subject of a recent review which concluded that although there is substantial evidence from pre-clinical studies showing the gut microbiota influence the physiological stress response further work in human populations was needed to realise the potential benefits of positively influencing the gut microbiota for the management of stress [5].

Probiotics (live microorganisms that when consumed in sufficient quantity confer a health benefit to the host) are one way in which the healthy balance of gut microbiota can be restored. They therefore offer an exciting opportunity to enhance psychological wellbeing and prevent stress. Previous studies have shown that probiotic supplementation can reduce symptoms of depression, anxiety and perceived stress in healthy volunteers although most of these studies are limited by small sample sizes [6]. In the Probiotics in Pregnancy (PIP) Study we were able to show that women who were supplemented with *Lactobacillus rhamnosus* HN001 had lower postnatal depression and anxiety scores than those on the placebo [7]. Furthermore the results showed that supplementation with *L. rhamnosus* HN001 improved anxiety and depression in all women not just in those who had high levels of depression or anxiety. This is shown in the two figures below. The significance of this finding is that it indicates that probiotic supplementation may be beneficial for the psychological wellbeing of all participants not simply those who already have high levels of stress, anxiety or depression.

University students experience increased stress associated with examinations. A study of Japanese medical students found that stress increased over an 8 week period prior to a national examination and peaked the day before the examination. Stress then decreased to baseline two weeks later. The same study found that those students supplemented with *Lactobacillus casei* (strain Shirota) had fewer gastrointestinal symptoms and cold and flu like symptoms [8, 9]. Similar results were reported in a study of stressed American university students where those supplemented with *Bifidobacterium bifdum* reported fewer days of cold and flu symptoms and more healthy days than those given placebo [10]. To date there has not been a large study of the effect of probiotic supplementation for psychological symptoms of stress, anxiety and mental wellbeing in university students.

**AIMS**

The overall aim of the PRESS Study is to investigate the effect of probiotic supplementation to reduce exam stress in university students. Specifically, the study aims to examine whether supplementation with the probiotic *Lactobacillus rhamnosus* HN001:

1. Reduces the build-up of stress experienced by university students leading up to examinations. This is the primary outcome of interest.
2. Reduces anxiety symptoms in students prior to examinations.
3. Improves psychological wellbeing of students prior to examinations.

**METHODS**

**Study design**

The PRESS Study is a randomised, double-blind, placebo-controlled trial with an allocation ratio of 1:1 intervention to placebo.

**Recruitment**

The New Zealand university system operates on two semesters per year with examinations for a paper held at the end of a semester. The PRESS Study will recruit 815 undergraduate students enrolled in Semester 1 of the 2020 university teaching year which begins on 2 March 2020. Advertisement of the study will be through placement of study information on individual online course pages and by providing invitation presentations at the beginning of lectures.

**Data Collection**

All consent and data collection will be managed by an online web-interface so students can register for the study and complete the questionnaires using their mobile phone, tablet, or computer.

**Exclusion criteria**

There are three exclusion criteria for the study. Participants will be excluded if they are:

- Currently taking probiotic supplement regularly
- Currently taking immunosuppressant’s e.g. chemotherapy
- Currently participating in another trial

**Sample Size**

To give a 90% chance of detecting a difference of 1.6 points (0.25 SD) in perceived stress scale scores between the probiotic supplemented group and placebo group at the 5% level we need 326 participants in each arm of the study. This equals a sample size of 652. Allowing for 25% attrition we need to recruit a total of 815 students.

**Measures**

There is one primary outcome of interest and two secondary outcomes. Self-reported perceived stress is the primary outcome while self-reported anxiety and psychological wellbeing are secondary outcomes of interest.

*Stress*

The Perceived Stress Scale is a 10 item questionnaire that asks about stress and coping in the previous month. Scores range from 0-40 with higher scores being indicative of higher levels of stress. Scores from 0-13 are considered to be low stress, 14-26= moderate stress and 27-40=high stress. In the PRESS pilot trial participating students had a mean PSS score of 22.1 (SD=6.3) at the end of the semester prior to their exam. Of the 105 respondents at this time point 9.5% reported low stress, 62.9% reported scores in the moderate range and 27.6% reported scores in the high range.

*Anxiety*

State Trait Anxiety Inventory 6 item version (STAI6): The STAI6 is a short 6 item scale validated as an anxiety screening questionnaire based on the longer State Trait Anxiety Inventory [11]. A cut-off of score >15 was used as an indicator of clinically significant levels of anxiety. In the PRESS pilot trial 59.1% reported anxiety scores in the high range at the end of semester prior to their exam. In the general population only 10% would be expected to report high anxiety scores.

*Psychological Well-Being*

The World Health Organisation well-being index the WHO-5 is a five item, positively worded measure of psychological well-being score ranging from 0 to 25. Higher scores represent better well-being. Scores of 13 or lower indicate low levels of psychological well-being. A systematic review of the WHO-5 concluded that it was a widely used and sensitive measure of depression [12] In the PRESS pilot trial 76.2% of participants reported low levels of psychological wellbeing at the end of semester before their exam.

**Intervention**

Capsules containing the probiotic *Lactobacillus rhamnosus* HN001 (6×10^9^ colony forming units) manufactured to pharmaceutical grade will be supplied by Fonterra Cooperative Group Limited. Placebo capsules identical in appearance and smell to the probiotic contain corn-derived maltodextrin. Both probiotic and placebo capsules are lactose free, gluten free, and contain no animal products.

*Safety*

The probiotic *L. rhamnosus* HN001 (6 X 10^9^ cfu) has been safely used in previous studies conducted in New Zealand including in pregnant women [7] and infants [13].

*Intervention period*

Students will take one capsule a day from the time that they are enrolled in the study and supplied with the capsules until three days prior to the commencement of examinations. The intervention period will be between 8-12 weeks but a minimum of 8 weeks.

*Randomisation*

Fonterra Cooperative Group Limited will manage the randomisation schedule which will be concealed from study staff and participants. A computer generated sequence will be used to randomise participants using a numbered system. Participants will be assigned a unique study number sequentially in the order that they register to participate in the study. Participants will be provided with a numbered bottle of capsules corresponding to their study number.

**Statistical Analysis**

An intention-to-treat analysis will be undertaken using SAS 9.4. The primary outcome of interest will be change in perceived stress from baseline to end of intervention. Two sample t-test will determine whether there is a statistically significant difference between the probiotic and placebo supplemented groups in change in perceived stress score. Similarly, for the two secondary outcomes, anxiety and psychological wellbeing, two sample t-tests will examine whether there is a significant difference between supplementation groups.

**Koha/Gift Vouchers**

To acknowledge the contribution of participants to this research, each participant will receive a $10 Munchy Mart voucher for completing the baseline questionnaires. Participants receive a further $15 Munchy Mart voucher for completing the end-of-intervention questionnaires. Munchy Mart is similar to a large dairy or 7/11 store on campus that stocks a wide range of snacks and lunch food at student prices.

**Ethical Approval**

Ethical approval from the University of Auckland Human Participants Ethics Committee will be obtained prior to commencement of recruitment.

*Informed consent*

All participants in this study will give full informed consent to their participation in the trial. Consent will be given using an electronic consent form.

*Data privacy*

All data collected as part of this trial will be securely held in electronic form on password protected folders within password protected servers. Only the researchers will have access to identifiable information during the study. All data will be deidentified prior to publication of any results in accordance with ethical guidelines.

**Trial registration**

The trial will be prospectively registered with the Australia and New Zealand Clinical Trials Registry

**REFERENCES**

1. Rea K, Dinan TG, Cryan JF (2016) The microbiome: A key regulator of stress and neuroinflammation. Neurobiology of Stress. doi: 10.1016/j.ynstr.2016.03.001

2. Bailey MT, Cryan JF (2017) The microbiome as a key regulator of brain, behavior and immunity: Commentary on the 2017 named series. Brain Behavior and Immunity. doi: 10.1016/j.bbi.2017.08.017

3. Foster JA, Rinaman L, Cryan JF (2017) Stress & the gut-brain axis: Regulation by the microbiome. Neurobiology of Stress. doi: 10.1016/j.ynstr.2017.03.001

4. Uday C Ghoshal (2018) Gut Microbiota-Brain Axis Modulation by a Healthier Microbiological Microenvironment : Facts and Fictions. Journal of Neurogastroenterology and Motility

5. Molina-Torres G, Rodriguez-Arrastia M, Roman P, Sanchez-Labraca N, Cardona D (2019) Stress and the gut microbiota-brain axis. Behavioural Pharmacology. doi: 10.1097/FBP.0000000000000478

6. McKean J, Naug H, Nikbakht E, Amiet B, Colson N (2017) Probiotics and Subclinical Psychological Symptoms in Healthy Participants: A Systematic Review and Meta-Analysis. J Altern Complement Med. doi: 10.1089/acm.2016.0023 [doi]

7. Slykerman RF, Hood F, Wickens K, Thompson JMD, Barthow C, Murphy R, Kang J, Rowden J, Stone P, Crane J, Stanley T, Abels P, Purdie G, Maude R, Mitchell EA (2017) Effect of Lactobacillus rhamnosus HN001 in Pregnancy on Postpartum Symptoms of Depression and Anxiety: A Randomised Double-blind Placebo-controlled Trial. EBioMedicine. doi: 10.1016/j.ebiom.2017.09.013

8. Kato-Kataoka A, Nishida K, Takada M, Kawai M, Kikuchi-Hayakawa H, Suda K, Ishikawa H, Gondo Y, Shimizu K, Matsuki T, Kushiro A, Hoshi R, Watanabe O, Igarashi T, Miyazaki K, Kuwano Y, Rokutan K (2016) Fermented Milk Containing Lactobacillus casei Strain Shirota Preserves the Diversity of the Gut Microbiota and Relieves Abdominal Dysfunction in Healthy Medical Students Exposed to Academic Stress. Appl Environ Microbiol. doi: 10.1128/AEM.04134-15 [doi]

9. Takada M, Nishida K, Kataoka‐Kato A, Gondo Y, Ishikawa H, Suda K, Kawai M, Hoshi R, Watanabe O, Igarashi T, Kuwano Y, Miyazaki K, Rokutan K (2016) Probiotic Lactobacillus casei strain Shirota relieves stress‐associated symptoms by modulating the gut–brain interaction in human and animal models. Neurogastroenterology & Motility. doi: 10.1111/nmo.12804

10. Langkamp-Henken B, Rowe CC, Ford AL, Christman MC, Nieves J, Carmelo, Khouri L, Specht GJ, Girard S, Spaiser SJ, Dahl WJ (2015) Bifidobacterium bifidum R0071 results in a greater proportion of healthy days and a lower percentage of academically stressed students reporting a day of cold/flu: a randomised, double-blind, placebo-controlled study. The British journal of nutrition. doi: 10.1017/S0007114514003997

11. Marteau TM, Bekker H (1992) The development of a six-item short-form of the state scale of the Spielberger State-Trait Anxiety Inventory (STAI). Br J Clin Psychol

12. Topp CW, Østergaard SD, Søndergaard S, Bech P (2015) The WHO-5 Well-Being Index: A Systematic Review of the Literature. Psychotherapy and Psychosomatics. doi: 10.1159/000376585

13. Wickens, Kristin, PhD|Black, Peter N., FRACP|Stanley, Thorsten V., FRCP|Mitchell, Edwin, FRACP, DSc|Fitzharris, Penny, FRACP|Tannock, Gerald W., PhD|Purdie, Gordon, BSc|Crane, Julian, FRACP (2008) A differential effect of 2 probiotics in the prevention of eczema and atopy: A double-blind, randomized, placebo-controlled trial. Journal of Allergy and Clinical Immunology, The. doi: 10.1016/j.jaci.2008.07.011

**FIGURES**

**Figure 1.** Cumulative frequency of State Trait Anxiety Inventory (STAI6)Scores for women taking *L. rhamnosus* HN001 and placebo in the Probiotics in Pregnancy Study

**Figure 2.** Cumulative frequency of Edinburgh Postnatal Depression Scale (EPDS) Scores for women taking *L. rhamnosus* HN001 and placebo in the Probiotics in Pregnancy Study.
