# Supplementary material for: Probiotics for Reduction of Examination Stress in Students (PRESS) study: A randomized, double-blind, placebo-controlled trial of the probiotic Lacticaseibacillus rhamnosus HN001
Source: PLoS One. 2022 Jun 8;17(6):e0267778. doi: 10.1371/journal.pone.0267778 (PMC9176810; doi:10.1371/journal.pone.0267778)
Supplement: Attachment — Submitted filename: renamed_5325c.docx [file pone.0267778.s004.docx]

**COMMENTS – Manuscript PONE-D-21-34328**

Title: “Probiotics for Reduction of Examination Stress in Students (PRESS) Study: A randomized, double-blind, placebo-controlled trial of the probiotic Lactobacillus rhamnosus HN001”

**Important note: This review pertains only to ‘statistical aspects’ of the study and so ‘clinical aspects’ [like medical importance, relevance of the study, ‘clinical significance and implication(s)’ of the whole study, etc.] are to be evaluated [should be assessed] separately/independently. Further please note that any ‘statistical review’ is generally done under the assumption that (such) study specific methodological [as well as execution] issues are perfectly taken care of by the investigator(s). This review is not an exception to that and so does not cover clinical aspects {however, seldom comments are made only if those issues are intimately / scientifically related & intermingle with ‘statistical aspects’ of the study}. Agreed that ‘statistical methods’ are used as just tools here, however, they are vital part of methodology [and so should be given due importance]. To improve the article/presentation, clues/hints may be taken from this review but should not limit the process by adhering to those points alone.**

**COMMENTS: Though the measures/tools used are appropriate [like Perceived Stress Scale, State Trait Anxiety Inventory, World Health Organisation - Five Well-Being Index] all/most of them yield data that are in [at the most] ‘ordinal’ level of measurement [and not in ratio level of measurement for sure {as the score two times higher does not indicate presence of that parameter/phenomenon as double (for example, a Visual Analogue Scales VAS score or say ‘depression’ score)}]. Then application of suitable non-parametric test(s) is/are indicated/advisable [even if distribution may be ‘Gaussian’ (i.e. normal)]. Agreed that there is/are no non-parametric test(s)/technique(s) available to be used as alternative in all situation(s) [suitable / most desired/applicable], but should be used whenever/wherever they are available. According to lines 32-33 {T-tests compared the change in psychological outcomes between groups} using parametric test(s) is not indicated/desired/correct. That may not change the direction of the result(s); however, correct methods are to be used always.**

**Moreover, remember that “Absence of evidence is not evidence of absence” [Altman DG, Bland JM. BMJ volume 311, 1995, p 485 (Reprinted : Australian Veterinary Journal 1996;74, 311)]. {Even when P-value is not significantly lower that is null hypothesis of no difference is not rejected, (in short, result is not significant), that does not amount to evidence of absence i.e. it does imply that there no difference. It only implies that there is no (i.e. these samples do not provide) enough evidence to prove (rather indicate with certain specified confidence level) the difference}.**

**Refer to lines 254 onwards [‘Explanation for the lack of a significant association between probiotic supplementation and psychological outcomes is that young adult university students in our sample may have had reduced adherence to the daily capsule intake], may not suffice. As said, adherence to medication is low in young adults compared with older age groups, even in the presence of chronic illness [26], may be true but trialist is/are expected to take steps/precautions to avoid or reduce this phenomenon {as for this manuscript ‘Article Type: Clinical Trial’ and not observational study}. Such excuse is not welcome or expected with respect a clinical trial in academic circles, in my experience.**

**Further, note that to provide a description of baseline characteristics is entirely reasonable (since it is clearly important in assessing to whom the results of the trial can be applied), however, it does not require the division of baseline characteristics by treatment groups (however, if done – alright). Statistical comparison of baseline characteristics [last ‘p-value’ column in Table 1: Participant characteristics by intervention group] is not desirable at all [because even if P-value turns out to be significant (while comparing baseline characteristics despite random allocation), it is, by definition, a false positive] as you then are supposed to be testing ‘randomization’ then, which in any single trial may not balance all baseline characteristics because ‘randomization’ is a sort of ‘insurance’ and not a guarantee scheme.**

References:

1. Stuart J. Pocock, et al., ‘Subgroup analysis, covariate adjustment and baseline comparisons in clinical trial reporting: current practice and problems’, Statistics in medicine, 2002; 21:2917–2930 [Particularly page 2927]
2. Harrington D, et al., ‘New guidelines for statistical reporting in the journal’, N Engl J Med 2019;381:285-6

[Important message (indirectly/ultimately indicated) from these articles: Never do any comparison with respect to ‘baseline’ characteristics {by applying statistical significance test(s)}, when allocation is done randomly].

**In fact, comparison with respect to ‘Post Intervention Scores’ should not be done unless you adjust for ‘baseline’ [may be ANCOVA]. ‘Change scores’ {Table 2: Mean change in stress, anxiety and psychological wellbeing scores between baseline to post-intervention for the probiotic and placebo groups} may be tested for significance by suitable ‘non-parametric’ test [say Mann-Whitney ‘U’ test].**

**Information given in lines 169-177 regarding the ‘Sample size’ is not as per the CONSORT guidelines. This is {**our final sample size gave a 90% chance of detecting a difference in Perceived Stress Scale scores of 2.9 points between the probiotic and placebo groups, at the 5% level of significance**} not the way to give the information required, in my opinion. What is required is it [required sample size for the study] was ‘estimated’ (namely the assumptions).**

**In my considered opinion, ‘let the respected editor decide the future course’.**
