# Supplementary material for: Probiotics for Reduction of Examination Stress in Students (PRESS) study: A randomized, double-blind, placebo-controlled trial of the probiotic Lacticaseibacillus rhamnosus HN001
Source: PLoS One. 2022 Jun 8;17(6):e0267778. doi: 10.1371/journal.pone.0267778 (PMC9176810; doi:10.1371/journal.pone.0267778)
Supplement: Attachment — Submitted filename: Response to Review itemised.docx [file pone.0267778.s005.docx]

We would like to thank the Academic Editor and the Reviewers for taking the time to review and comment on our manuscript. Please find below an itemised response to each comment detailing how we have made changes to the manuscript in response to each point raised.

**Response to Academic Editorial Comments**

1. When submitting your revision, we need you to address these additional requirements.

Please ensure that your manuscript meets PLOS ONE's style requirements, including those for file naming. The PLOS ONE style templates can be found at 
[https://journals.plos.org/plosone/s/file?id=wjVg/PLOSOne_formatting_sample_main_body.pdf](https://protect-au.mimecast.com/s/rLFVCL7Ep5FLLy34FmmW7z?domain=journals.plos.org) and 
[https://journals.plos.org/plosone/s/file?id=ba62/PLOSOne_formatting_sample_title_authors_affiliations.pdf](https://protect-au.mimecast.com/s/dudZCMwGq5hjjMJWfQdWOX?domain=journals.plos.org)

**Response**: Thank you for including these links we have edited the manuscript to comply with the PLOS ONE formatting and style requirements.

2. Thank you for stating the following in the Acknowledgments Section of your manuscript: 
(This study was funded by Fonterra Cooperative Group Limited)
We note that you have provided funding information that is not currently declared in your Funding Statement. However, funding information should not appear in the Acknowledgments section or other areas of your manuscript. We will only publish funding information present in the Funding Statement section of the online submission form. 
Please remove any funding-related text from the manuscript and let us know how you would like to update your Funding Statement. Currently, your Funding Statement reads as follows: 
(This project received funding from Fonterra Cooperative Group Limited.)
Please include your amended statements within your cover letter; we will change the online submission form on your behalf.

**Response:** We have removed the funding statement from the Acknowledgements section of the manuscript. The correct funding statement should read “This study received funding from Fonterra Cooperative Group Limited. Grant number 5000855.

3. We note that you have indicated that data from this study are available upon request. PLOS only allows data to be available upon request if there are legal or ethical restrictions on sharing data publicly. For more information on unacceptable data access restrictions, please see [http://journals.plos.org/plosone/s/data-availability#loc-unacceptable-data-access-restrictions](https://protect-au.mimecast.com/s/_mZ0CNLJr5tooD6Xf0TPFJ?domain=journals.plos.org).

In your revised cover letter, please address the following prompts:

a) If there are ethical or legal restrictions on sharing a de-identified data set, please explain them in detail (e.g., data contain potentially sensitive information, data are owned by a third-party organization, etc.) and who has imposed them (e.g., an ethics committee). Please also provide contact information for a data access committee, ethics committee, or other institutional body to which data requests may be sent.

b) If there are no restrictions, please upload the minimal anonymized data set necessary to replicate your study findings as either Supporting Information files or to a stable, public repository and provide us with the relevant URLs, DOIs, or accession numbers. For a list of acceptable repositories, please see [http://journals.plos.org/plosone/s/data-availability#loc-recommended-repositories](https://protect-au.mimecast.com/s/qYjnCOMKv5h33zO4iAwDzl?domain=journals.plos.org).

We will update your Data Availability statement on your behalf to reflect the information you provide.

- **Response:** We have now uploaded a deidentified dataset as Supporting Information in the online submission form. Thank you for the offer of updating our Data Availability Statement, to save you this task we have amended the data availability statement in the manuscript file and in the online submission form. We have used the suggested wording *All relevant data are within the manuscript and its Supporting Information files.”*

4. Please include captions for your Supporting Information files at the end of your manuscript, and update any in-text citations to match accordingly. Please see our Supporting Information guidelines for more information: [http://journals.plos.org/plosone/s/supporting-information](https://protect-au.mimecast.com/s/FidWCP7Lw5FQQ86MCZ7tWV?domain=journals.plos.org).

**Response**: Thank you we have added a heading Supporting Information and a caption for the file at the end of the manuscript

5. Thank you for submitting the above manuscript to PLOS ONE. During our internal evaluation of the manuscript, we found significant text overlap between your submission and the following previously published work, of which you are an author.

- [https://www.ANZCTR.org.au/Steps11and12/379312-(Uploaded-20-02-2020-07-02-22)-Study-related%20document.docx](https://protect-au.mimecast.com/s/m2SkCQnMx5C77MRrfGQttq?domain=anzctr.org.au)

We would like to make you aware that copying extracts from previous publications, especially outside the methods section, word-for-word is unacceptable. In addition, the reproduction of text from published reports has implications for the copyright that may apply to the publications.

Please revise the manuscript to rephrase the duplicated text, cite your sources, and provide details as to how the current manuscript advances on previous work. Please note that further consideration is dependent on the submission of a manuscript that addresses these concerns about the overlap in text with published work.

We will carefully review your manuscript upon resubmission, so please ensure that your revision is thorough.

**Response:** Thank you for bringing this issue to our attention. The file you reference which contains duplicate information is the trial protocol that was uploaded to the Australia and New Zealand Clinical Trials Registry prior to the commencement of the clinical trial. This is not a published work. Changing the wording of the manuscript is likely to decrease the quality of the written work. To address any potential copyright issues for the journal we have elected to replace the trial protocol that appears on the trial registry with a summary protocol that has re-written the background information section so as not to overlap with this manuscript. We have had confirmation from the ANZCTR that the protocol file has been updated and I have checked the registry to ensure the previous file which contained duplicate wording has been removed.

While revising your submission, please upload your figure files to the Preflight Analysis and Conversion Engine (PACE) digital diagnostic tool, [https://pacev2.apexcovantage.com/](https://protect-au.mimecast.com/s/PmWvCXLWGktmmV90iLMbyl?domain=pacev2.apexcovantage.com). PACE helps ensure that figures meet PLOS requirements. To use PACE, you must first register as a user. Registration is free. Then, login and navigate to the UPLOAD tab, where you will find detailed instructions on how to use the tool. If you encounter any issues or have any questions when using PACE, please email PLOS at [figures@plos.org](mailto:figures@plos.org). Please note that Supporting Information files do not need this step.

**Response**: Thank you we have now uploaded our figure into the PACE system.

**Response to Reviewer comments**

**Reviewer #1:** The clinical trial developed by Slykerman et al is relevant, but I have some concerns.

It's unclear why the authors chose the Rhamnosus strain. Since in the introduction the authors report benefits of several probiotic strains.

**Response**: We chose the rhamnosus strain based on our previous work with this strain in clinical trials. In the Probiotics in Pregnancy Study we found pregnant women who were supplemented with HN001 had significantly lower postnatal depression and anxiety. This is explained in lines 90-100 of the introduction. We have added to the introduction in this section to clarify that this previous work provides a rationale for using this strain in the current study.
In my opinion there is a concern about adherence to treatment by students.
How can authors be sure students take the capsule daily?

**Response:** We agree that adherence is an issue, particularly in younger people. During the trial, participants received regular text messages to their mobile phone reminding them to take the capsules which we have now noted in the manuscript. We cannot be sure that every participant took the capsules daily and we have therefore discussed this as a limitation of the study in the discussion section. We used an intent-to-treat analysis approach to take into account the realities of conducting trials in human populations.

Treatment time is unclear too. How many days did the students take the capsules? Time is a determining factor in the result.

**Response:** Thank you for raising this point, we have added to the manuscript to clarify treatment time in the methods section. Treatment time was a minimum of 8 weeks and up to 13 weeks depending on when participants enrolled in the study. In the discussion we have acknowledged treatment time and discussed this point. Variation in treatment time would be expected to be similar in both groups (probiotic and placebo) given randomisation of participants. The majority of reviewed studies in Pirbeglou et al. had intervention periods of less than 8 weeks and a minimum intervention period of 8 weeks in our study would not be out of alignment with other clinical trials in the field.

In conclusion authors state: "difficulty of translating preclinical evidence". However, the authors cite several clinical trials showing the strain's efficacy for anxiety and stress (see references 15, 16 and 7 and 8).
I think adherence to treatment and time can interfere with the results.

**Response**: Thank you for this comment regarding the conclusion statement in the abstract. One of the difficulties of conducting clinical trials in human populations is managing issues such as adherence. We have altered the conclusion to reflect that adherence is one of the reasons why translating pre-clinical evidence into human populations is challenging. In addition, large macro-level events such as the COVID19 pandemic can also alter the expected trajectory of psychological wellbeing in research participants therefore impacting results. The conclusion in the abstract is now in-line with the conclusion in the manuscript.

**Reviewer #2:** Important note: This review pertains only to ‘statistical aspects’ of the study and so ‘clinical aspects’ [like medical importance, relevance of the study, ‘clinical significance and implication(s)’ of the whole study, etc.] are to be evaluated [should be assessed] separately/independently. Further please note that any ‘statistical review’ is generally done under the assumption that (such) study specific methodological [as well as execution] issues are perfectly taken care of by the investigator(s). This review is not an exception to that and so does not cover clinical aspects {however, seldom comments are made only if those issues are intimately / scientifically related & intermingle with ‘statistical aspects’ of the study}. Agreed that ‘statistical methods’ are used as just tools here, however, they are vital part of methodology [and so should be given due importance]. To improve the article/presentation, clues/hints may be taken from this review but should not limit the process by adhering to those points alone.

COMMENTS: Though the measures/tools used are appropriate [like Perceived Stress Scale, State Trait Anxiety Inventory, World Health Organisation - Five Well-Being Index] all/most of them yield data that are in [at the most] ‘ordinal’ level of measurement [and not in ratio level of measurement for sure {as the score two times higher does not indicate presence of that parameter/phenomenon as double (for example, a Visual Analogue Scales VAS score or say ‘depression’ score)}]. Then application of suitable non-parametric test(s) is/are indicated/advisable [even if distribution may be ‘Gaussian’ (i.e. normal)]. Agreed that there is/are no non-parametric test(s)/technique(s) available to be used as alternative in all situation(s) [suitable / most desired/applicable], but should be used whenever/wherever they are available. According to lines 32-33 {T-tests compared the change in psychological outcomes between groups} using parametric test(s) is not indicated/desired/correct. That may not change the direction of the result(s); however, correct methods are to be used always.

**Response:** Thank you for this comment regarding the analytical approach. To address this comment we have now reported the median and interquartile range for stress, anxiety, and psychological wellbeing in Table 1 replacing the mean and standard deviation. We have also conducted the Mann Whitney U test as suggested by the Reviewer for non-parametric data. We have updated Table 2 to show these results. As the Reviewer suggested this has not changed the direction of the results.

Moreover, remember that “Absence of evidence is not evidence of absence” [Altman DG, Bland JM. BMJ volume 311, 1995, p 485 (Reprinted : Australian Veterinary Journal 1996;74, 311)]. {Even when P-value is not significantly lower that is null hypothesis of no difference is not rejected, (in short, result is not significant), that does not amount to evidence of absence i.e. it does imply that there no difference. It only implies that there is no (i.e. these samples do not provide) enough evidence to prove (rather indicate with certain specified confidence level) the difference}.

**Response:** We have reviewed the manuscript and made changes to the wording of the discussion section to clarify that the results of our study do not provide evidence to reject the null hypothesis. We have taken care to ensure that we have clarified in the discussion that we did not find a *statistically significant* difference between the groups.

Refer to lines 254 onwards [‘Explanation for the lack of a significant association between probiotic supplementation and psychological outcomes is that young adult university students in our sample may have had reduced adherence to the daily capsule intake], may not suffice. As said, adherence to medication is low in young adults compared with older age groups, even in the presence of chronic illness [26], may be true but trialist is/are expected to take steps/precautions to avoid or reduce this phenomenon {as for this manuscript ‘Article Type: Clinical Trial’ and not observational study}. Such excuse is not welcome or expected with respect a clinical trial in academic circles, in my experience.

**Response:** We sent regular text messages to participants during the intervention to remind them to take their capsules and encourage adherence. It is very difficult to measure adherence to treatment when the treatment is capsules. Counting capsules and self-report questionnaires are not reliable. Due to COVID19 lockdown restrictions and stay at home orders, participants did not attend regular in-person study appointments. Ideally, we would have used Medication Event Monitoring System (MEMS) which records the date, time, and frequency of vial openings, however this was beyond the resources of this RCT. This is a limitation, but we have used intention to treat analysis, and one would expect that lack of adherence would affect each treatment group equally.

Further, note that to provide a description of baseline characteristics is entirely reasonable (since it is clearly important in assessing to whom the results of the trial can be applied), however, it does not require the division of baseline characteristics by treatment groups (however, if done – alright). Statistical comparison of baseline characteristics [last ‘p-value’ column in Table 1: Participant characteristics by intervention group] is not desirable at all [because even if P-value turns out to be significant (while comparing baseline characteristics despite random allocation), it is, by definition, a false positive] as you then are supposed to be testing ‘randomization’ then, which in any single trial may not balance all baseline characteristics because ‘randomization’ is a sort of ‘insurance’ and not a guarantee scheme.
References:
1. Stuart J. Pocock, et al., ‘Subgroup analysis, covariate adjustment and baseline comparisons in clinical trial reporting: current practice and problems’, Statistics in medicine, 2002; 21:2917–2930 [Particularly page 2927]
2. Harrington D, et al., ‘New guidelines for statistical reporting in the journal’, N Engl J Med 2019;381:285-6
[Important message (indirectly/ultimately indicated) from these articles: Never do any comparison with respect to ‘baseline’ characteristics {by applying statistical significance test(s)}, when allocation is done randomly].

**Response:** In light of this comment from the Reviewer we have replaced Table 1 with a new table in accordance with their suggestions. The table now describes the total sample of participants in this study.

In fact, comparison with respect to ‘Post Intervention Scores’ should not be done unless you adjust for ‘baseline’ [may be ANCOVA]. ‘Change scores’ {Table 2: Mean change in stress, anxiety and psychological wellbeing scores between baseline to post-intervention for the probiotic and placebo groups} may be tested for significance by suitable ‘non-parametric’ test [say Mann-Whitney ‘U’ test].

**Response:** We have now conducted the Mann Whitney U analyses and replaced Table 2 with an updated table.

Information given in lines 169-177 regarding the ‘Sample size’ is not as per the CONSORT guidelines. This is {our final sample size gave a 90% chance of detecting a difference in Perceived Stress Scale scores of 2.9 points between the probiotic and placebo groups, at the 5% level of significance} not the way to give the information required, in my opinion. What is required is it [required sample size for the study] was ‘estimated’ (namely the assumptions).

**Response:** Thank you for drawing this to our attention. We have reviewed the CONSORT guidelines on reporting of sample size. We have added to the manuscript to describe the initial target sample size based on a clinically meaningful reduction in stress scores and described this initial calculation.
